# Predictors of Return Visits Among Insured Emergency Department Mental Health and Substance Abuse Patients, 2005–2013

**DOI:** 10.5811/westjem.2017.6.33850

**Published:** 2017-07-17

**Authors:** Sangil Lee, Jeph Herrin, William V. Bobo, Ryan Johnson, Lindsey R. Sangaralingham, Ronna L. Campbell

**Affiliations:** * The University of Iowa Carver College of Medicine, Department of Emergency Medicine, Iowa City, Iowa; † Yale University School of Medicine, Department of Cardiology, New Havens, Connecticut; ‡ Health Research & Educational Trust, Chicago, Illinois; § Mayo Clinic, Department of Psychiatry, Rochester, Minnesota; ¶ Robert D. and Patricia E. Kern Center for the Science of Healthcare Delivery, Mayo Clinic, Rochester, Minnesota; || Mayo Clinic, Department of Emergency Medicine, Rochester, Minnesota

## Abstract

**Introduction:**

Our goal was to describe the pattern and identify risk factors of early-return ED visits or inpatient admissions following an index mental health and substance abuse (MHSA)-related ED visit in the United States.

**Methods:**

We performed a retrospective cohort study using Optum Labs Data Warehouse, a nationally representative database containing administrative claims data on privately insured and Medicare Advantage enrollees. Authors identified patients presenting to an ED with a primary diagnosis of MHSA between 2005 and 2013 who were discharged home. Study inclusion required continuous insurance enrollment for the 12 months preceding and the 31 days following the index ED visit. During the study period we included only the first ED visit for each patient.

**Results:**

A total of 49,672 (14.2%) had a return visit to the ED or had a hospitalization within 30 days following discharge. Mean time to the next ED visit or inpatient admission was 11.7 days. An increased age (age 65+ vs. age <18 years; OR 1.65, 95% CI [1.57 to 1.74]), chronic medical comorbidities (Hwang comorbidity 5+ vs 0; OR 1.31, 95% CI [1.27 to 1.35]), prior ED and inpatient utilization (4+ visits vs 0 visits; OR 5.59, 95% CI [5.41 to 5.78]) were associated with return visits within 30 days following discharge.

**Conclusion:**

In an analysis of nearly 350,000 ED visits for MHSA, 14.2 % of patients returned to the ED or hospital within 30 days. This study identified a number of factors associated with return visits for acute care.

## INTRODUCTION

It is estimated that one in five Americans suffer from a chronic mental health and substance abuse (MHSA) issue,^1^ many of which require acute care. Reduction in the number of inpatient psychiatric beds since the 1960s has led to large increases in mental health-related emergency department (ED) visits in the United States.^2^ MHSA-related ED visits increased from an estimated 4–6% in 1992 to an estimated 12% of ED visits in the U.S. in 2007.^3^–^5^ EDs are frequently used for the initial evaluation of MHSA emergencies and many patients treated in the ED for MHSA return soon for acute care; a previous study of ED return visits related to pediatric psychiatric illness from Canada indicated that as many as 15% of patients with mental illness returned to the ED within three days. 6 Thus, return visits for acute care following the initial ED visit may represent avoidable healthcare utilization.

Currently, little is known about the characteristics of patients who present to the ED for psychiatric care, and, more importantly, who among these patients are at high risk for early return for acute care whether mental health related or not. Elucidating the trend, timing of return beyond the three-day mark, and risk factors for return visits may enable clinicians to develop strategies for preventing early-return visits for acute care and assist policymakers with appropriate resource allocation for those at highest risk for early return.

We used a national insurance-claims database to describe the trend and identify risk factors of return visits to the ED or hospitalization occurring within 3, 7 and 30 days after index ED discharge for MHSA evaluation. The primary objective was to identify risk factors for early-return ED visits.

## METHODS

### Data Source

We conducted an analysis using the Optum Labs Data Warehouse (OLDW), a database including administrative claims on privately insured and Medicare Advantage enrollees.^7^ OLDW has been used previously in studies of the therapeutic patterns and outcomes of patient care.^7^–^9^ The database includes all medical claims for over 100 million enrollees throughout the U.S.^7^, ^10^ Medical claims and enrollment files include information on birth year, sex, dates of enrollment coverage, *International Classification of Diseases, 9th Revision, Clinical Modification* (ICD-9-CM) diagnosis codes, ICD-9 procedure codes, *Current Procedural Terminology, Version 4* (CPT-4) procedure codes, *Healthcare Common Procedure Coding System* (HCPCS) procedure codes, place of service codes and provider specialty codes. We accessed study data in compliance with the Health Insurance Portability and Accountability Act of 1996. This study involved only the analysis of pre-existing, de-identified data and was therefore exempt from institutional review board approval.

### Selection of Participants

We identified all ED visits of patients presenting to the ED with a primary diagnosis of a MHSA-related visit between January 1, 2005, and November 30, 2013. To be included in the cohort, patients were required to have had continuous medical coverage for at least 12 months prior to the index ED visit and 31 days after the visit. We defined 12 months of continuous coverage as the requirement to ensure the reliability of longitudinal data, which is similar to the previous study using OLDW.^10^ ED visits related to MHSA conditions were determined by using the primary ICD-9-CM diagnosis codes. Agency for Healthcare Research and Quality (AHRQ) Clinical Classifications Software (CCS) for ICD-9-CM categorization scheme was applied to categorize the primary diagnosis for each index ED visit (Supplemental Table 1). 11 CCS is the software that collapses the ICD-9 code into a smaller number of clinically useful categories.^11^

Population Health Research Capsule

What do we already know about this issue?

Emergency Departments are frequently used for the evaluation of mental health and substance abuse related emergencies. Many patients treated in the ED for MHSA return soon for acute care.

What was the research question?

To describe the trend and identify risk factors of return visits to the ED visit occurring after index ED discharge for MHSA evaluation.

What was the major finding of the study?

In an analysis of ED visits for mental health and substance abuse, 14.2 % of patients returned to the ED or hospital within 30 days with several risk factors for returns, including age, previous utilization and comorbidities.

How does this improve population health?

Our findings may help to elucidate the group that may benefit from intense outpatient referral to prevent unnecessary return visits.

We excluded ED visits if there was a hospital admission on the same or next day of ED discharge. ED visits were also excluded if they had a primary diagnosis that fell into the CCS categories for delirium, dementia, other amnestic disorders, and developmental disorder, as there are substantial overlaps with medical evaluation and admission associated with these diagnoses. We also excluded ED visits if there was a MHSA-related ED visit within the prior 12 months in order to set a wash-out period and accurately define the index visit. Without a visit-free period before the index visit, we would have needed to characterize not only factors on presentation, but also factors known from prior visits, as well as the trajectory of prior visits. By requiring a visit-free period, we were able to infer that the risk factors for return could be isolated to the index visit. If a patient had more than one qualifying ED visit over the study period, we considered the earliest ED visit as their index visit.

### Independent Variables

Risk factors for ED return visits or hospitalization were chosen a priori based on expert opinion and literature review. 6, 12–14 Covariates included age group (< 18, 18–35, 36–64, and 65+ years), sex, number of chronic conditions classified using the Hwang index (0, 1, 2, 3, 4, 5+), 15 race/ethnicity (White, African-American, Hispanic, Asian) non-mental health related ED utilization within 12 months prior to the index ED visit (0, 1, 2, 3, 4+ encounters), and MHSA as primary diagnosis (Supplemental Table 1). The Hwang comorbidity method is a sum of chronic conditions, with a higher count being associated with increased comorbidity burden. 15 The comorbid conditions were identified by ICD-9-CM codes in the primary or secondary diagnosis on any claim during the 12-month baseline period. We used an additional Hwang index of 5+ to account for a high proportion of medical comorbidities.

### Outcomes

Our main outcome of interest was a return ED visit or inpatient admission occurring within 30 days of discharge from an index ED visit. Follow-up began on the day following the index ED visit, and ended on the date of a return visit to the ED, the date of a psychiatric or non-psychiatric hospitalization, or 31 days following discharge from an index ED visit, whichever occurred first. We classified return ED visits or hospitalizations as being for MHSA purposes by the presence of a primary diagnosis included in CCS categories for MHSA as identified above; other return ED visits or hospitalizations were classified as being for non-mental conditions. We counted a return ED visit or hospitalization as study endpoints only if they occurred on days 2–30 following the date of discharge from an index ED visit; as noted above, admissions the next day resulted in exclusion of the ED visit from the cohort due to frequent extended ED stay, while next ED admissions were considered part of the same ED visit. We looked at three nested outcome events: those occurring within 2–3 days, within 2–7 days, and within 2–30 days.

### Analysis

We summarized the demographic and clinical characteristics of the cohort using means and proportions, as appropriate. The frequencies of each risk factor were compared between the groups characterized by the study outcomes (return ED visit or hospitalization at 3, 7 and 30 days) using chi-squared tests. Then, to assess the independent association of each risk factor with return visits, we estimated a multiple logistic regression model for each outcome (3-day, 7-day, and 30-day return to acute care), incorporating all risk factors as predictor variables. For categorical variables we reported adjusted odds ratios (OR), and we reported C-statistics for all logistic regression models. Outcomes of the multiple logistic regression and time-to-event analyses were reported using OR with 95% confidence intervals (CI). We determined statistical significance by p-values < 0.05. All analyses were performed using SAS 9.3 (SAS Institute, Cary NC, USA, 2014) and Stata 14 (StataCorp, College Station TX, 2015).

## RESULTS

### Characteristics of Study Subjects

We identified a total of 350,406 qualifying index ED visits as the cohort (Figure 1). Demographic and descriptive characteristics of the cohort members are shown in Table 1. The cohort consisted primarily of non-geriatric adults, the majority of whom were White (52.0%) and female (54.4%). The majority of patients had at least one co-occurring chronic medical condition. Nearly 60% of cohort members had no evidence of ED utilization in the year preceding the index ED visit. The most common mental health conditions at the index ED visits were alcohol and other substance use disorders, anxiety disorders, and mood disorders (Table 1).

### Main Results

Among the index ED visits during 2005–2013, 3.1% (n=10,860) of patients returned to the ED or were hospitalized within three days, 6.1% (n=21,348) within seven days, and 14.2% (n=49,672) within 30 days after the index ED visit cumulatively (Figure 1). The mean ± standard deviation (SD) time to early ED return visits or hospitalization was 11.7 (SD 8.6 days). Supplemental Figure 1 shows the distribution of the return visits. Among the 43,572 ED return visits, 17, 249 (39.6%) were due to MHSA conditions, and among the 6,100 hospitalizations, 4,542 (74.5%) were due to MHSA conditions.

Table 1 and Supplemental Table 1a, 1b show the comparative frequencies of early-return ED visits or hospitalization within 3, 7, and 30 days for each covariate, using those without early return to ED or hospitalization as a control group. The risk of early-return ED visits or hospitalization within 3 days, 7 days, and 30 days was associated with age, sex, race, Hwang index, and prior ED utilization and multiple CCS diagnostic categories. Because of the large number of patients with unknown race/ethnicity information, this variable was excluded from the models.

The results of the multiple logistic regression analyses are shown in Table 2, and supplemental tables 2a, 2b. Male sex was associated with an increased odds of early-return ED visit or hospitalization within three days (OR 1.09, 95% CI [1.05 to 1.13]), 7 days (OR 1.09, 95% CI [1.06 to 1.12]), and 30 days (30 days OR 1.08; 95% CI [1.06 to1.10]) (Table 2). Increasing age (age 65+ years vs. age <18 years; OR 1.65; 95% CI [1.57 to 1.74]), increasing medical comorbidity (Hwang comorbidity 5+ vs. 0; OR 1.31; 95% CI [1.27 to 1.35]), and prior ED utilization (4+ visits vs 0 visits; OR 5.59; 95% CI 5.[41 to 5.78]) were also associated with return visits within 30 days (Table 2). Similar results were found for early-return ED visits or hospitalization at three and seven days (Table 2, and supplemental tables 2a, 2b). The C-statistics ranged from 0.66–0.69 for the MHSA-related and all models, and 0.71–0.72 for the non-MHSA models.

Primary MHSA diagnosis of personality disorders (OR 1.59, 95% CI [1.35 to1.87]), schizophrenia and other psychotic disorders (OR 1.31, 95% CI [1.24 to 1.39]), mood disorders (OR 1.28, 95% CI [1.23 to1.34]), and substance abuse (OR1.14, 95%CI 1.09 to 1.20) were associated with increased odds of early-return ED visit or hospitalization within 30 days. Similar findings were observed for early-return ED visits or hospitalizations at three days and at seven days (Table 2). Alcohol-related disorders (OR 0.61, 95% CI [0.58 to 0.64]), anxiety disorders (OR 0.88, 95% CI [0.84 to 0.92]) and suicide and intentional self-inflicted injury (OR 0.91, 95% CI [0.84 to 0.99]) were associated with significantly decreased odds of early-return ED visit or hospitalization within 30 days. Similar findings were observed for the odds of early-return ED visit or hospitalization at three days and at seven days (Table 2, and supplemental tables 2a, 2b).

## DISCUSSION

### Principal Findings

This large, U.S.-based retrospective analysis of over 350,000 patients evaluated a population of individuals with continuous medical coverage for 12 months, presenting to the ED for a MHSA (excluding delirium, dementia, amnestic disorders, or developmental disorders) for the first time in at least 12 months and whose ED visit did not result in a hospitalization, to assess the rate of return to the ED within the next 30 days for either mental health or non-mental health reasons. The study showed a high rate (14.2%) of early-return ED visits or hospitalizations within 30 days of index discharge. For those who sought acute care within 30 days of index discharge, the median time to the next utilization was nearly nine days. Increased age, comorbidity burden, prior acute care utilization, diagnosed personality disorders, schizophrenia (and related psychoses), mood disorders and substance abuse were associated with an increased odds of return ED visits or hospitalization within 3, 7 and 30 days of index discharge.

### Comparison with Prior Studies

The overall rates of return within 3 days, 7 days, and 30 days in our cohort were 3.1%, 6.1%, and 14.2%, respectively. This is consistent with the results of Pham et al., who used the National Hospital Ambulatory Medical Care Survey and reported a three-day general ED return rate of 3.2% for all ages and diagnoses. 13 In contrast, Rising et al. reported general ED return visits rates of 7.5% within three days and 22.4% within 30 days.^16^ The increased rates reported by Rising et al. may be due to the fact that only adult patients were included. 16 Since the rate of return visits due to a MHSA condition seems to be increasing steadily, we propose that outpatient follow-up within one to two weeks might be a reasonable strategy for those who are at high risk for return ED visits after a MHSA-related evaluation.

We found that the median time to a return visit was nine days. Our results are consistent with those of Rising et al. who studied patterns of early ED return visits using data from the AHRQ Cost and Utilization Project and reported that the most frequent time interval to an early ED return visit was about nine days. 16 Although our study was limited to mental health-related visits and included both ED return visits and hospitalizations, the latter of which may or may not have a second associated ED visit, these results suggest that the commonly used metric of a three-day return rate is capturing only a small portion of potentially avoidable healthcare delivery among patients with MHSA-related conditions.

The highest rates of both MHSA-related and non-MHSA-related ED return visits at 3, 7, and 30 days in our cohort were in patients aged 65 years and older (Table 2). Increasing age and medical comorbidity burden were also identified as independent predictors of the risk for early return for acute care in our study. The absence of overlaps in the ORs and 95% CI suggested that the increased age and comorbidities were more associated with non-MHSA returns than MHSA returns. Martin-Gill et al. reported that both increased age and the presence of mental illness were associated with increased rates of 72-hour return visits among general ED patients.^17^ Gabayan et al. used the California Office of Statewide Health Planning and Development files to examine the general geriatric population presenting to the ED and reported an increased hospitalization rate in those with a mental illness diagnosis (OR 2.17).^12^

Prior ED utilization was found to be one of the strongest risk factors for return visits in our study. A previous study of geriatric patients demonstrated that patients with prior utilization were more likely to return to the ED. 18 Our study showed that previous non mental health-related ED utilization independently predicted early return visits after an index mental health-related ED visit. Prior ED utilization may be related to patient functional status, limited primary care access or other factors.

With regard to specific MHSA disorders, diagnosed personality disorders, schizophrenia, mood disorders and substance abuse were identified as significant risk factors for return visits to acute care, consistent with prior literature. 6, 14, 19–21 Hesling et al. reported lower utilization of home healthcare after discharge for depression or schizophrenia compared to non-MHSA conditions. 22 These are some of the frequent MHSAs seen in the ED, and may benefit from specific interventions, such as expedited outpatient referral within 1–2 weeks and arrangement for home health visits for those who cannot. 22, 23

Somewhat surprisingly, alcohol use disorders and suicide and intentional self-inflicted injury were inversely related with return visit in our study. One prior study showed lack of a significant relationship between the use of alcohol and negative health outcomes or treatment costs in inpatient settings. 24 Part of the reason for differences between our results and those of prior investigations may be our exclusion of patients who required inpatient admission at the time of the index ED visit. As such, our cohort may have been less ill than those of prior studies. This study’s findings imply that the chance of an ED return visit could increase when there is any mental health condition, particularly depression, anxiety or substance abuse-related conditions, that requires some type of intervention. If clinicians or healthcare policymakers want to decrease the unnecessary utilization by allowing an easier access to outpatient resources, whether it be access to outpatient clinics or crisis units before the expected time to return, in our study, it would be within nine days.

## LIMITATIONS

Our study has several limitations. First, it is observational, and as with any observational study any associations may be biased by unobserved confounders; at the same time, we can’t make any causal inferences from the associations. Second, this cohort represents a commercially insured population that we assumed may experience different and fewer socioeconomic stressors than uninsured or underinsured populations. Because the data derived from a private insurance population and 12 months of enrollment status was required, the results likely under-represented the magnitude of ED utilization and return visits. This is particularly important for persons with severe or persistent mental disorders, such as schizophrenia or other psychotic disorders, and severe mood disorders, a substantial number of whom rely on publically-funded insurance programs, or are uninsured and frequently present to acute healthcare settings in need of urgent treatment for psychiatric and non-psychiatric problems. 22 Third, there was a substantial amount of unreported data for variables that are important factors in healthcare utilization, particularly race, income, availability of psychiatrists in the ED, access to primary care, size of healthcare system, psychiatric bed capacity, availability of assertive community treatment, family support and other intensive community-based MHSA treatment programs. Additional research with complementary or alternate datasets should be conducted to examine the influence of these factors. 25 Related to this, we did not have mortality data on our cohort; patients who expired outside the acute care setting during the 30 days post-ED visit could not be counted. Lastly, we did not include the index ED visit resulting in a hospitalization, as our primary interest was to evaluate the trend of ED return visits after discharge from the ED.

## CONCLUSION

In summary, an analysis of over 350,000 ED visits for mental health treatment over eight years indicated that 14.2% of patients returned to acute care within 30 days of index ED discharge. Older age and prior ED utilization were the strongest risk factors for early return to acute care, both for MHSA and non-MHSA reasons. Additional risk factors for early return to acute care were also observed. Furthermore, the decline in inpatient psychiatry bed capacity might have contributed to the increase in mental health-related ED visits. It is time to explore creative solutions to improve care for MHSA conditions after ED evaluation. Our findings may help to elucidate the group that could benefit from intense outpatient referral to prevent unnecessary return visits.

## Supplementary Information













## Figures and Tables

**Figure:**
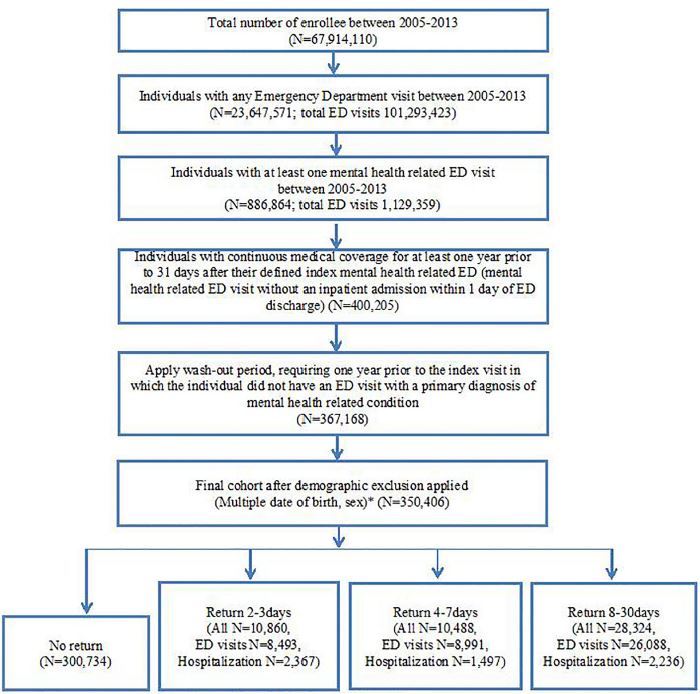
Flow chart of selection process. *ED*, emergency department. N=Total number of patients.

**Table 1:** Demographic data and rates of 3 day, 7 day, and 30 day return ED visit or hospital admission.

	Characteristics	All returns			MHSA returns		
			
		3 day	7 day	30 day	3 day	7 day	30 day
Variable	N* (%)	N (%)	N (%)	N (%)	N (%)	N (%)	N (%)
N	35,0406(100.0)	10,860(100.0)	21,348(100.0)	49,672(100.0)	63,12(100.0)	11,326(100.0)	21,791(100.0)
Age mean (SD)	36(17.6)						
Age (category)							
<18	50,446(14.4)	989(9.1)	2072(9.7)	5,422(10.9)	718(11.4)	1,446(12.8)	3,293(15.1)
18–35	134,695(38.4)	3,470(32.0)	6,761(31.7)	16,049(32.3)	2,084(33.0)	3,661(32.3)	7,113(32.6)
36–64	142,586(40.7)	5,166(47.6)	10,082(47.2)	22,671(45.6)	2,911(46.1)	5,203(45.9)	9,628(44.2)
>65	22,679 (6.5)	1,235(11.4)	2,433(11.4)	5,530(11.1)	599(9.5)	1,016(9.0)	1,757(8.1)
Sex							
Female	190,461(54.4)	5,941(54.7)	11,773( 55.1)	27,739(55.8)	3,295(52.2)	5,924(52.3)	11,335(52.0)
Male	159,945(45.6)	4,919(45.3)	9,575( 44.9)	21,933(44.2)	3,017(47.8)	5,402(47.7)	10,456(48.0)
Race/ethnicity							
Caucasian	182,103(52.0)	5,897(54.3)	11,449( 53.6)	26,641(53.6)	3,505(55.5)	6,204(54.8)	11,915(54.7)
Hispanic	27,754(7.9)	721(6.6)	1,412( 6.6)	3,380(6.8)	369(5.8)	644(5.7)	1,280(5.9)
African American	24,818(7.1)	883(8.1)	1,800( 8.4)	4,330(8.7)	466(7.4)	855(7.5)	1,616(7.4)
Asian	5,888(1.7)	140(1.3)	2,66( 1.2)	596(1.2)	86(1.4)	143(1.3)	280(1.3)
Unknown	109,843(31.3)	3,219(29.6)	6,421( 30.1)	14,725(29.6)	1,886(29.9)	3,480(30.7)	6,700(30.7)
Hwang group							
0	723,62(20.7)	2,201(20.3)	4,218( 19.8)	10,160(20.5)	1,209(19.2)	2,059(18.2)	4,001(18.4)
1	703,61(20.1)	1,498(13.8)	2,861( 13.4)	6,542(13.2)	920(14.6)	1,585(14.0)	3,107(14.3)
2	63,639(18.2)	1,591(14.7)	3,214( 15.1)	7,496(15.1)	993(15.7)	1,907(16.8)	3,805(17.5)
3	47,990(13.7)	1,515(14.0)	2,919( 13.7)	6,795(13.7)	959(15.2)	1,715(15.1)	3,396(15.6)
4	32,183(9.2)	1,114(10.3)	2,216( 10.4)	5,025(10.1)	704(11.2)	1,285(11.3)	2,433(11.2)
5+	63,871(18.2)	2,941(27.1)	5,920( 27.7)	13,654(27.5)	1,527(24.2)	2,775(24.5)	5,049(23.2)
Prior EDs							
0	203,657(58.1)	4,425 (40.7)	8,411( 39.4)	18,881(38.0)	2,904(46.0)	5,163(45.6)	9,983(45.8)
1	78,194(22.3)	2,500(23.0)	4,874( 22.8)	11,456(23.1)	1,512(24.0)	2,709(23.9)	5,272(24.2)
2	31,642(9.0)	1,328(12.2)	2,660( 12.5)	6,349(12.8)	740(11.7)	1,329(11.7)	2,576(11.8)
3	14,764(4.2)	786(7.2)	1,612 (7.6)	3,878(7.8)	417(6.6)	763(6.7)	1,427(6.5)
4+	22,149(6.3)	18,21(16.8)	3,791(17.8)	9,108(18.3)	739(11.7)	1,362(12.0)	2,533(11.6)
Initial visit CCS category*							
Adjustment							
No		10,653(98.1)	20,936( 98.1)	48,666(98.0)	6,175(97.8)	11,065(97.7)	21,295(97.7)
Yes	6,480(1.8)	207(1.9)	412 ( 1.9)	1006(2.0)	137(2.2)	261(2.3)	496(2.3)
Anxiety							
No		7,199(66.3)	13,773( 64.5)	31,522(63.5)	4,579(72.5)	8,001(70.6)	15,294(70.2)
Yes	130,828(37.3)	3,661(33.7)	7,575( 35.5)	18,150(36.5)	1,733(27.5)	3,325(29.4)	6,497(29.8)
ADHD							
No		10,572(97.3)	20,778( 97.3)	48,362(97.4)	6,096(96.6)	10,911(96.3)	20,974(96.3)
Yes	9,452(2.7)	288(2.7)	570 ( 2.7)	1310(2.6)	216(3.4)	415(3.7)	817( 3.7)
D/O childhood							
No		10,824 (99.7)	21,277(99.7)	49,479(99.6)	6,288(99.6)	11,276(99.6)	21,667(99.4)
Yes	1,390(0.4)	36(0.3)	71(0.3)	193(0.4)	24(0.4)	50(0.4)	124(0.6)
Impulse							
No		10,843(99.8)	21,317(99.9)	49,573(99.8)	6,299(99.8)	11,302(99.8)	21,722(99.7)
Yes	665(0.2)	17(0.2)	31(0.1)	99(0.2)	13(0.2)	24(0.2)	69(0.3)
Mood							
No		8,195(75.5)	16,187(75.8)	3,7901(76.3)	4,310(68.3)	7,737(68.3)	14,945(68.6)
Yes	64,657(18.5)	2,665 (24.5)	5,161(24.2)	11,771(23.7)	2,002(31.7)	3,589(31.7)	6,846(31.4)
Personality							
No		10,809(99.5)	21,252(99.6)	49,456(99.6)	6,277(99.4)	11,261(99.4)	21,660(99.4)
Yes	953(0.3)	51(0.5)	96(0.4)	216(0.4)	35(0.6)	65(0.6)	131(0.6)
Schizophrenia							
No		9,815(90.4)	19,463( 91.2)	45,839(92.3)	5,664(89.7)	10,278(90.7)	20,047(92.0)
Yes	16,821(4.8)	1,045(9.6)	1,885(8.8)	3,833(7.7)	648(10.3)	1,048(9.3)	1,744(8.0)
Alcohol							
No		9,278(85.4)	18,356(86.0)	42,612(85.8)	5,332(84.5)	9,626(85.0)	18,360(84.3)
Yes	86,427(24.7)	1,582(14.6)	2,992 ( 14.0)	7,060( 14.2)	980(15.5)	1,700(15.0)	3,431(15.7)
Substance							
No		9,364(86.2)	18,516(86.7)	43,366(87.3)	5,453(86.4)	9,891(87.3)	19,214(88.2)
Yes	35,308(10.1)	1,496(13.8)	2,832(13.3)	6,306(12.7)	859(13.6)	1,435(12.7)	2,577(11.8)
Suicide*							
No		10,702(98.5)	21,061(98.7)	48,904(98.5)	6,197(92.2)	11,135(98.3)	21,387(98.1)
Yes	5,749(1.6)	158(1.5)	287(1.3)	768(1.5)	115(1.8)	191(1.7)	404(1.9)
Screening							
No		10,655(98.1)	20,921(98.0)	48,648(97.9)	6,217(98.5)	11,147(98.4)	21,407(98.2)
Yes	6,239(1.8)	205(1.9)	427(2.0)	1024(2.1)	95(1.5)	179(1.6)	384(1.8)
Miscellaneous							
No		10,512(96.8)	20,618(96.6)	47,957(96.5)	6,209(98.4)	11,115(98.1)	21,382(98.1)
Yes	9,196(2.6)	348(3.2)	730(3.4)	1715(3.5)	103 (1.6)	211(1.9)	409(1.9)

*CCS,* clinical classifications software; *ED*, emergency department; *MHSA*, mental health and substance abuse; *SD*, standard deviation.

*ADHD*, Attention-deficit, conduct, and disruptive behavior disorders; *D/O childhood*, disorders usually diagnosed in infancy, childhood, or adolescence.

* Each visit may contain multiple primary diagnosis codes that fall in different CCS categories

* Suicide ideation and attempt

* N= Total

**Table 2:** Logistic regression analysis showing rates of 3 day, 7 day, and 30 day return ED visit or hospital admission by patient characteristics.

	All returns			MHSA returns		
		
	3day	7day	30day	3day	7day	30day
Characteristic	OR	OR	OR	OR	OR	OR
Sex						
Female	ref	ref	Ref	Ref	ref	Ref
Male	1.09 [1.05,1.13]	1.09 [1.06,1.12]	1.08 [1.06,1.10]	1.14 [1.08,1.20]	1.16 [1.11,1.20]	1.18 [1.14,1.21]
Age (category)						
<18	ref	ref	Ref	Ref	ref	Ref
18–35	1.41 [1.30,1.52]	1.28 [1.22,1.35]	1.14 [1.10,1.18]	1.31 [1.19,1.43]	1.13 [1.06,1.21]	0.94 [0.90,0.99]
36–64	1.76 [1.63,1.90]	1.59 [1.51,1.68]	1.34 [1.30,1.39]	1.58 [1.45,1.74]	1.38 [1.29,1.47]	1.11 [1.06,1.16]
>65	2.02 [1.83,2.22]	1.85 [1.72,1.98]	1.65 [1.57,1.74]	1.64 [1.45,1.86]	1.37 [1.25,1.51]	1.06 [0.99,1.14]
Hwang						
0	Ref	Ref	Ref	Ref	ref	ref
1	0.95 [0.89,1.02]	0.96 [0.91,1.01]	0.89 [0.86,0.93]	0.99 [0.90,1.08]	0.98 [0.92,1.05]	0.96 [0.91,1.01]
2	0.99 [0.93,1.06]	1.06 [1.00,1.11]	1.02 [0.99,1.06]	1.03 [0.94,1.12]	1.15 [1.07,1.22]	1.16 [1.11,1.22]
3	1.15 [1.07,1.23]	1.17 [1.11,1.23]	1.15 [1.11,1.20]	1.22 [1.12,1.34]	1.28 [1.19,1.36]	1.31 [1.24,1.37]
4	1.16 [1.07,1.25]	1.22 [1.16,1.29]	1.19 [1.14,1.24]	1.27 [1.15,1.39]	1.36 [1.26,1.46]	1.36 [1.28,1.43]
5+	1.18 [1.11,1.25]	1.27 [1.21,1.33]	1.31 [1.27,1.35]	1.15 [1.06,1.25]	1.26 [1.18,1.34]	1.25 [1.20,1.31]
Prior EDs						
0	Ref	Ref	Ref	Ref	ref	ref
1	1.38 [1.31,1.45]	1.42 [1.37,1.48]	1.55 [1.51,1.59]	1.27 [1.20,1.36]	1.29 [1.23,1.36]	1.33 [1.29,1.38]
2	1.73 [1.62,1.84]	1.86 [1.78,1.95]	2.15 [2.08,2.22]	1.49 [1.37,1.62]	1.53 [1.44,1.63]	1.60 [1.53,1.68]
3	2.12 [1.96,2.30]	2.39 [2.26,2.53]	2.93 [2.82,3.06]	1.74 [1.57,1.94]	1.85 [1.71,2.00]	1.90 [1.79,2.02]
4+	3.24 [3.05,3.44]	3.89 [3.72,4.06]	5.59 [5.41,5.78]	2.03 [1.86,2.21]	2.21 [2.07,2.36]	2.32 [2.21,2.44]
Initial CCS category						
Adjustment						
No	Ref	Ref	Ref	Ref	ref	Ref
Yes	1.12 [0.96,1.30]	1.11 [1.00,1.24]	1.09 [1.01,1.18]	1.30 [1.09,1.56]	1.37 [1.20,1.57]	1.27 [1.15,1.40]
Anxiety						
No	Ref	Ref	Ref	Ref	ref	Ref
Yes	0.91 [0.84,0.99]	0.94 [0.89,1.00]	0.88 [0.84,0.92]	0.83 [0.75,0.92]	0.88 [0.82,0.95]	0.84 [0.79,0.89]
ADHD						
No	Ref	Ref	Ref	Ref	ref	Ref
Yes	1.38 [1.20,1.59]	1.32 [1.19,1.46]	1.14 [1.06,1.22]	1.75 [1.49,2.06]	1.74 [1.55,1.96]	1.50 [1.37,1.63]
D/O Childhood						
No	Ref	Ref	Ref	Ref	ref	Ref
Yes	1.22 [0.87,1.72]	1.16 [0.90,1.48]	1.19 [1.01,1.39]	1.32 [0.87,2.00]	1.40 [1.05,1.88]	1.53 [1.26,1.86]
Impulse						
No	Ref	Ref	Ref	Ref	ref	Ref
Yes	1.03 [0.63,1.68]	0.92 [0.63,1.32]	1.16 [0.93,1.45]	1.33 [0.76,2.31]	1.29 [0.86,1.95]	1.76 [1.36,2.27]
Mood						
No	Ref	Ref	Ref	Ref	ref	Ref
Yes	1.44 [1.33,1.56]	1.40 [1.32,1.48]	1.28 [1.23,1.34]	2.11 [1.93,2.32]	2.10 [1.96,2.25]	1.96 [1.86,2.06]
Personality						
No	Ref	Ref	Ref	Ref	ref	Ref
Yes	1.82 [1.35,2.44]	1.70 [1.36,2.12]	1.59 [1.35,1.87]	2.08 [1.47,2.96]	2.05 [1.58,2.67]	1.97 [1.62,2.39]
Schizophrenia						
No	Ref	Ref	Ref	Ref	ref	Ref
Yes	1.74 [1.57,1.91]	1.59 [1.47,1.71]	1.31 [1.24,1.39]	2.20 [1.95,2.47]	2.02 [1.84,2.21]	1.71 [1.59,1.83]
Alcohol						
No	Ref	Ref	Ref	Ref	ref	Ref
Yes	0.71 [0.64,0.77]	0.67 [0.63,0.72]	0.61 [0.58,0.64]	0.81 [0.73,0.91]	0.80 [0.73,0.87]	0.77 [0.72,0.82]
Substance						
No	Ref	Ref	Ref	Ref	ref	Ref
Yes	1.37 [1.25,1.50]	1.30 [1.22,1.39]	1.14 [1.09,1.20]	1.56 [1.39,1.74]	1.45 [1.33,1.58]	1.26 [1.18,1.34]
Suicide						
No	Ref	Ref	Ref	Ref	ref	Ref
Yes	0.95 [0.80,1.12]	0.85 [0.75,0.96]	0.91 [0.84,0.99]	1.15 [0.95,1.40]	1.03 [0.89,1.20]	1.06 [0.96,1.18]
Screening						
No	Ref	Ref	Ref	Ref	ref	Ref
Yes	1.05 [0.90,1.22]	1.10 [0.99,1.23]	1.06 [0.98,1.15]	0.91 [0.74,1.13]	0.97 [0.82,1.13]	1.02 [0.91,1.14]
Miscellaneous						
No	Ref	Ref	Ref	Ref	ref	Ref
Yes	1.16 [1.02,1.32]	1.21 [1.10,1.33]	1.11 [1.04,1.18]	0.70 [0.57,0.86]	0.79 [0.68,0.92]	0.73 [0.65,0.82]

*CCS,* clinical classifications software; *ED*, emergency department; *MHSA*, mental health and substance abuse; *ADHD*, Attention-deficit, conduct, and disruptive behavior disorders; *D/O childhood*, disorders usually diagnosed in infancy, childhood, or adolescence.
