# Supplementary material for: Predictors of Return Visits Among Insured Emergency Department Mental Health and Substance Abuse Patients, 2005–2013
Source: West J Emerg Med. 2017 Jul 17;18(5):884–93. doi: 10.5811/westjem.2017.6.33850 (PMC5576625; doi:10.5811/westjem.2017.6.33850)
Supplement: Supplementary file 1 [file wjem-18-884-s001.docx]

Supplemental Table 1: CCS categories for mental health conditions (abbreviation for tables).

| 650 | Adjustment disorders (adjustment) |
| --- | --- |
| 651 | Anxiety disorders (anxiety) |
| 652 | Attention-deficit, conduct, and disruptive behavior disorders (ADHD) |
| 655 | Disorders usually diagnosed in infancy, childhood, or adolescence (D/O Childhood) |
| 656 | Impulse control disorders (impulse) |
| 657 | Mood disorders (mood) |
| 658 | Personality disorders (personality) |
| 659 | Schizophrenia and other psychotic disorders (schizophrenia) |
| 660 | Alcohol-related disorders (alcohol) |
| 661 | Substance-related disorders (substance) |
| 662 | Suicide and intentional self-inflicted injury (suicide) |
| 663 | Screening and history of mental health and substance abuse codes (screening) |
| 670 | Miscellaneous disorders (miscellaneous) |
