# Supplementary material for: Predictors of Return Visits Among Insured Emergency Department Mental Health and Substance Abuse Patients, 2005–2013
Source: West J Emerg Med. 2017 Jul 17;18(5):884–93. doi: 10.5811/westjem.2017.6.33850 (PMC5576625; doi:10.5811/westjem.2017.6.33850)
Supplement: Supplementary file 2 [file wjem-18-884-s002.docx]

**Supplemental Table 1a.** Rates of 3 day, 7 day, and 30 day return ED visits by patient characteristics.

|  |  | |  |  |  |  |  |  |  |
| --- | --- | --- | --- | --- | --- | --- | --- | --- | --- |
|  |  | |  | **All returns** |  |  | **MHSA Returns** | |  |
|  |  | | **3 day** | **7 day** | **30 day** | **3 day** | **7 day** | **30 day** |  |
| **Variable** |  | | **n (%)** | **n (%)** | **n (%)** | **n (%)** | **n (%)** | **n (%)** |  |
|  | | | 8493 (100.0) | 17484 (100.0) | 43572 (100.0) | 4278 (100.0) | 8134 (100.0) | 17249 (100.0) |  |
| **Age (category)** | | |  |  |  |  |  |  |  |
|  | <18 | | 700 ( 8.2) | 1543 ( 8.8) | 4538 ( 10.4) | 441 ( 10.3) | 947 ( 11.6) | 2472 ( 14.3) |  |
|  | 18-35 | | 2729 ( 32.1) | 5610 ( 32.1) | 14272 (32.8) | 1407 ( 32.9) | 2636 ( 32.4) | 5658 ( 32.8) |  |
|  | 36-64 | | 4021 ( 47.3) | 8195 ( 46.9) | 19694 (45.2) | 1977 ( 46.2) | 3729 ( 45.8) | 7579 ( 43.9) |  |
|  | >65 | | 1043 ( 12.3) | 2136 ( 12.2) | 5068 (22.6) | 453 ( 10.6) | 822 ( 10.1) | 1540 ( 8.9) |  |
| **Sex** |  | |  |  |  |  |  |  |  |
|  | Female | | 4726 ( 55.6) | 9782 ( 55.9) | 24568 (56.4) | 2287 ( 53.5) | 4340 ( 53.4) | 9082 ( 52.7) |  |
|  | Male | | 3767 ( 44.4) | 7702 ( 44.1) | 19004 (43.6) | 1991 ( 46.5) | 3794 ( 46.4) | 8167 ( 47.3) |  |
| **Race/Ethnicity** |  | |  |  |  |  |  |  |  |
|  | Caucasian | | 4603 ( 54.2) | 9339 ( 53.4) | 23257 (53.4) | 2391 ( 55.9) | 4439 ( 54.6) | 9364 ( 54.3) |  |
|  | Hispanic | | 592 ( 7.0) | 1201 ( 6.9) | 3062 (7.0) | 260 ( 6.1) | 477 ( 5.9) | 1055 ( 6.1) |  |
|  | African American | | 723 ( 8.5) | 1530 ( 8.8) | 3913 (9.0) | 329 ( 7.7) | 637 ( 7.8) | 1321 ( 7.7) |  |
|  | Asian | | 105 ( 1.2) | 215 ( 1.2) | 524 ( 1.2) | 54 ( 1.3) | 99 ( 1.2) | 224 ( 1.3) |  |
|  | Unknown | | 2470 ( 29.1) | 5199 ( 29.7) | 12816 (29.4) | 1244 ( 29.1) | 2482 ( 30.5) | 5285 ( 30.6) |  |
| **Hwang Group** | | |  |  |  |  |  |  |  |
|  | | 0 | 1781 ( 21.0) | 3554 ( 20.3) | 9148 (21.0) | 833 ( 19.5) | 1477 ( 18.2) | 3188 ( 18.5) |  |
|  | | 1 | 1216 ( 14.3) | 2450 ( 14.0) | 5875 ( 13.5) | 664 ( 15.5) | 1224 ( 15.0) | 2560 ( 14.8) |  |
|  | | 2 | 1212 ( 14.3) | 2589 ( 14.8) | 6519 (15.0) | 658 ( 15.4) | 1366 ( 16.8) | 3002 ( 17.4) |  |
|  | | 3 | 1148 ( 13.5) | 2319 ( 13.3) | 5832 (13.4) | 628 ( 14.7) | 1187 ( 14.6) | 2606 ( 15.1) |  |
|  | | 4 | 819 ( 9.6) | 1729 ( 9.9) | 4293 (9.9) | 456 ( 10.7) | 886 ( 10.9) | 1892 ( 11.0) |  |
|  | | 5+ | 2317 ( 27.3) | 4843 ( 27.7) | 11905 (27.3) | 1039 ( 24.3) | 1994 ( 24.5) | 4001 ( 23.2) |  |
| **Prior EDs** | |  |  |  |  |  |  |  |  |
|  | | 0 | 3319 ( 39.1) | 6615 ( 37.8) | 16037 ( 36.8) | 1918 ( 44.8) | 3611 ( 44.4) | 7728 ( 44.8) |  |
|  | | 1 | 1919 ( 22.6) | 3965 ( 22.7) | 9957 (22.9) | 1023 ( 23.9) | 1970 ( 24.2) | 4154 ( 24.1) |  |
|  | | 2 | 1027 ( 12.1) | 2167 ( 12.4) | 5586 (12.8) | 485 ( 11.3) | 938 ( 11.5) | 2047 ( 11.9) |  |
|  | | 3 | 636 ( 7.5) | 1357 ( 7.8) | 3482 (8.0) | 294 ( 6.9) | 560 ( 6.9) | 1156 ( 6.7) |  |
|  | | 4+ | 1592 ( 18.7) | 3380 (19.3) | 8510 (19.5) | 558 ( 13.0) | 1055 ( 13.0) | 2164 ( 12.5) |  |
| **Initial visit CCS category** | | |  |  |  |  |  |  |  |
| **Adjustment** | | |  |  |  |  |  |  |  |
|  | No | | 8315 ( 97.9) | 17121 ( 97.9) | 42646 (97.9) | 4169 ( 97.5) | 7919 ( 97.4) | 16819 ( 97.5) |  |
|  | Yes | | 178 ( 2.1) | 363 ( 2.1) | 926 (2.1) | 109 ( 2.5) | 215 ( 2.6) | 430 ( 2.5) |  |
| **Anxiety** |  | |  |  |  |  |  |  |  |
|  | No | | 5251 ( 61.8) | 10683 ( 61.1) | 26824 (61.6) | 2838 ( 66.3) | 5313 ( 65.3) | 11516 ( 66.8) |  |
|  | Yes | | 3242 ( 38.2) | 6801 ( 38.9) | 16748 (38.4) | 1440 ( 33.7) | 2821 ( 34.7) | 5733 ( 33.2) |  |
| **ADHD** |  | |  |  |  |  |  |  |  |
|  | No | | 8292 ( 97.6) | 17067 ( 97.6) | 42488 (97.5) | 4146 ( 96.9) | 7859 ( 96.6) | 16634 ( 96.4) |  |
|  | Yes | | 201 ( 2.4) | 417 ( 2.4) | 1084 (2.5) | 132 ( 3.1) | 275 ( 3.4) | 615 ( 3.6) |  |
| **D/O Childhood** | | |  |  |  |  |  |  |  |
|  | No | | 8464 ( 99.7) | 17433 ( 99.7) | 43416 (99.6) | 4260 ( 99.6) | 8103 ( 99.6) | 17157 ( 99.5) |  |
|  | Yes | | 29 ( 0.3) | 51 ( 0.3) | 156 (0.4) | 18 ( 0.4) | 31 ( 0.4) | 92 ( 0.5) |  |
| **Impulse** |  | |  |  |  |  |  |  |  |
|  | No | | 8481 ( 99.9) | 17459 ( 99.9) | 43489 (99.8) | 4270 ( 99.8) | 8116 ( 99.8) | 17195 ( 99.7) |  |
|  | Yes | | 12 ( 0.1) | 25 ( 0.1) | 83 (0.2) | 8 ( 0.2) | 18 ( 0.2) | 54 ( 0.3) |  |
| **Mood** |  | |  |  |  |  |  |  |  |
|  | No | | 6638 ( 78.2) | 13540 ( 77.4) | 33593 (77.1) | 3045 ( 71.2) | 5676 ( 69.8) | 11951 ( 69.3) |  |
|  | Yes | | 1855 ( 21.8) | 3944 ( 22.6) | 9979 (22.9) | 1233 ( 28.8) | 2458 ( 30.2) | 5298 ( 30.7) |  |
| **Personality** | | |  |  |  |  |  |  |  |
|  | No | | 8455 ( 99.6) | 17411 ( 99.6) | 43387 (99.6) | 4255 ( 99.5) | 8092 ( 99.5) | 17148 ( 99.4) |  |
|  | Yes | | 38 ( 0.4) | 73 ( 0.4) | 185 (0.4) | 23 ( 0.5) | 42 ( 0.5) | 101 (0.6) |  |
| **Schizophrenia** | | |  |  |  |  |  |  |  |
|  | No | | 7790 ( 91.7) | 16087 ( 92.0) | 40367 (92.6) | 3918 ( 91.6) | 7480 ( 92.0) | 15958 ( 92.5) |  |
|  | Yes | | 703 ( 8.3) | 1397 ( 8.0) | 3205 (7.4) | 360 ( 8.4) | 654 ( 8.0) | 1291 ( 7.5) |  |
| **Alcohol** |  | |  |  |  |  |  |  |  |
|  | No | | 7319 ( 86.2) | 15209 ( 87.0) | 37672 (86.5) | 3661 ( 85.6) | 7053 ( 86.7) | 14744 ( 85.5) |  |
|  | Yes | | 1174 ( 13.8) | 2275 ( 13.0) | 5900 ( 13.5) | 617 ( 14.4) | 1081 ( 13.3) | 2505 ( 14.5) |  |
| **Substance** | | |  |  |  |  |  |  |  |
|  | No | | 7365 ( 86.7) | 15273 ( 87.4) | 38257 (87.8) | 3730 ( 87.2) | 7217 ( 88.7) | 15450 ( 89.6) |  |
|  | Yes | | 1128 ( 13.3) | 2211 ( 12.6) | 5315 (12.2) | 548 ( 12.8) | 917 ( 11.3) | 1799 ( 10.4) |  |
| **Suicide** |  | |  |  |  |  |  |  |  |
|  | No | | 8405 ( 99.0) | 17291 ( 98.9) | 42937 (98.5) | 4230 ( 98.9) | 8029 ( 98.7) | 16959 ( 98.3) |  |
|  | Yes | | 88 ( 1.0) | 193 ( 1.1) | 635 (1.5) | 48 ( 1.1) | 105 ( 1.3) | 290 ( 1.7) |  |
| **Screening** | | |  |  |  |  |  |  |  |
|  | No | | 8326 ( 98.0) | 17126 ( 98.0) | 42664 (97.9) | 4215 ( 98.5) | 8009 ( 98.5) | 16944 ( 98.2) |  |
|  | Yes | | 167 ( 2.0) | 358 ( 2.0) | 908 (2.1) | 63 ( 1.5) | 125 ( 1.5) | 305 ( 1.8) |  |
| **Miscellaneous** | | |  |  |  |  |  |  |  |
|  | No | | 8174 ( 96.2) | 16811 ( 96.2) | 41965 (96.3) | 4194 ( 98.0) | 7958 ( 97.8) | 16891 ( 97.9) |  |
|  | Yes | | 319 ( 3.8) | 673 ( 3.8) | 1607 (3.7) | 84 ( 2.0) | 176 ( 2.2) | 358 ( 2.1) |  |
