# Supplementary material for: Predictors of Return Visits Among Insured Emergency Department Mental Health and Substance Abuse Patients, 2005–2013
Source: West J Emerg Med. 2017 Jul 17;18(5):884–93. doi: 10.5811/westjem.2017.6.33850 (PMC5576625; doi:10.5811/westjem.2017.6.33850)
Supplement: Supplementary file 3 [file wjem-18-884-s003.docx]

**Supplemental Table 1b.** Rates of 3 day, 7 day, and 30 day return hospitalization by patient characteristics.

|  |  |  | **All returns** | |  | **MHSA Returns** | |
| --- | --- | --- | --- | --- | --- | --- | --- |
|  |  | **3 day** | **7 day** | **30 day** | **3 day** | **7 day** | **30 day** |
| **Variable** |  | **n (%)** | **n (%)** | **n (%)** | **n (%)** | **n (%)** | **n (%)** |
|  | | 2367 (100.0) | 3864 (100.0) | 6100 (100.0) | 2034 (100.0) | 3192 (100.0) | 4542 (100.0) |
| **Age (category)** | |  |  |  |  |  |  |
|  | <18 | 289 ( 12.2) | 529 ( 13.7) | 884 ( 14.5) | 277 ( 13.6) | 499 ( 15.6) | 821 ( 18.1) |
|  | 18-35 | 741 ( 31.3) | 1151 ( 29.8) | 1777 ( 29.1) | 677 ( 33.3) | 1025 ( 32.1) | 1455 ( 32.0) |
|  | 36-64 | 1145 ( 48.4) | 1887 ( 48.8) | 2977 ( 48.8) | 934 ( 45.9) | 1474 ( 46.2) | 2049 ( 45.1) |
|  | >65 | 192 ( 8.1) | 297 ( 7.7) | 462 ( 7.6) | 146 ( 7.2) | 194 ( 6.1) | 217 ( 4.8) |
| **Sex** |  |  |  |  |  |  |  |
|  | Female | 1215 ( 51.3) | 1991 ( 51.5) | 3171 ( 52.0) | 1008 ( 49.6) | 1584 ( 49.6) | 2253 ( 49.6) |
|  | Male | 1152 ( 48.7) | 1873 ( 48.5) | 2929 ( 48.0) | 1026 ( 50.4) | 1608 ( 50.4) | 2289 ( 50.4) |
| **Race/Ethnicity** |  |  |  |  |  |  |  |
|  | Caucasian | 1294 ( 54.7) | 2110 ( 54.6) | 3384 ( 55.5) | 1114 ( 54.8) | 1765 ( 55.3) | 2551 ( 56.3) |
|  | Hispanic | 129 ( 5.4) | 211 ( 5.5) | 318 ( 5.2) | 109 ( 5.4) | 167 ( 5.2) | 225 ( 5.0) |
|  | African American | 160 ( 6.8) | 270 ( 7.0) | 417 ( 6.8) | 137 ( 6.7) | 218 ( 6.8) | 295 ( 6.5) |
|  | Asian | 35 ( 1.5) | 51 ( 1.3) | 72 ( 1.2) | 32 ( 1.6) | 44 ( 1.4) | 56 ( 1.2) |
|  | Unknown | 749 ( 31.6) | 1222 ( 31.6) | 1909 ( 31.3) | 642 ( 31.6) | 998 ( 31.3) | 1415 ( 31.2) |
| **Hwang Group** | |  |  |  |  |  |  |
|  | 0 | 420 ( 17.7) | 664 ( 17.2) | 1012 ( 16.6) | 376 ( 18.5) | 582 ( 18.2) | 813 ( 17.9) |
|  | 1 | 282 ( 11.9) | 411 ( 10.6) | 667 ( 10.9) | 256 ( 12.6) | 361 ( 11.3) | 547 ( 12.0) |
|  | 2 | 379 ( 18.2) | 625 ( 16.2) | 977 ( 16.0) | 335 ( 16.5) | 541 ( 16.9) | 803 ( 17.7) |
|  | 3 | 367 ( 15.5) | 600 ( 15.5) | 963 ( 15.8) | 331 ( 16.3) | 528 ( 16.5) | 790 ( 17.4) |
|  | 4 | 295 ( 12.5) | 487 ( 12.6) | 732 ( 12.0) | 248 ( 12.2) | 399 ( 12.5) | 541 ( 11.9) |
|  | 5+ | 624 ( 26.4) | 1077 ( 27.9) | 1749 ( 28.7) | 488 ( 24.0) | 781 ( 24.5) | 1048 ( 23.1) |
| **Prior EDs** |  |  |  |  |  |  |  |
|  | 0 | 1106 ( 46.7) | 1796 ( 46.5) | 2844 ( 46.6) | 986 ( 48.5) | 1552 ( 48.6) | 2255 ( 49.6) |
|  | 1 | 581 ( 24.5) | 909 ( 23.5) | 1499 ( 24.6) | 489 ( 24.0) | 739 ( 23.2) | 1118 ( 24.6) |
|  | 2 | 301 ( 12.7) | 493 ( 12.8) | 763 ( 12.5) | 255 ( 12.5) | 391 ( 12.2) | 529 ( 11.6) |
|  | 3 | 150 ( 6.3) | 255 ( 6.6) | 396 ( 6.5) | 123 ( 6.0) | 203 ( 6.4) | 271 ( 6.0) |
|  | 4+ | 229 ( 9.7) | 411 ( 10.6) | 598 ( 9.8) | 181 ( 8.9) | 307 ( 9.6) | 369 ( 8.1) |
| **Initial visit CCS category** | |  |  |  |  |  |  |
| **Adjustment** | |  |  |  |  |  |  |
|  | No | 2338 ( 98.8) | 3815 ( 98.7) | 6020 ( 98.7) | 2006 ( 98.6) | 3146 ( 98.6) | 4476 ( 98.5) |
|  | Yes | 29 ( 1.2) | 49 ( 1.3) | 80 ( 1.3) | 28 ( 1.4) | 46 ( 1.4) | 66 ( 1.5) |
| **Anxiety** |  |  |  |  |  |  |  |
|  | No | 1948 ( 82.7) | 3090 ( 80.0) | 4698 ( 77.0) | 1741 ( 85.6) | 2688 ( 84.2) | 3778 ( 83.2) |
|  | Yes | 419 ( 17.7) | 774 ( 20.0) | 1402 ( 23.0) | 293 ( 14.4) | 504 ( 15.8) | 764 ( 16.8) |
| **ADHD** |  |  |  |  |  |  |  |
|  | No | 2280 ( 96.3) | 3711 ( 96.0) | 5874 ( 96.3) | 1950 ( 95.9) | 3052 ( 95.6) | 4340 ( 95.6) |
|  | Yes | 87 ( 3.7) | 153 ( 4.0) | 226 ( 3.7) | 84 ( 4.1) | 140 ( 4.4) | 202 ( 4.4) |
| **D/O Childhood** | |  |  |  |  |  |  |
|  | No | 2360 ( 99.7) | 3844 ( 99.5) | 6063 ( 99.4) | 2028 ( 99.7) | 3173 ( 99.4) | 4510 ( 99.3) |
|  | Yes | 7 ( 0.3) | 20 ( 0.5) | 37 ( 0.6) | 6 ( 0.3) | 19 ( 0.6) | 32 ( 0.7) |
| **Impulse** |  |  |  |  |  |  |  |
|  | No | 2362 ( 99.8) | 3858 ( 99.8) | 6084 ( 99.7) | 2029 ( 99.8) | 3186 ( 99.8) | 4527 ( 99.7) |
|  | Yes | 5 ( 0.2) | 6 ( 0.2) | 16 ( 0.3) | 5 ( 0.2) | 6 ( 0.2) | 15 ( 0.3) |
| **Mood** |  |  |  |  |  |  |  |
|  | No | 1557 ( 65.8) | 2647 ( 68.5) | 4308 ( 70.6) | 1265 ( 62.2) | 2061 ( 64.6) | 2994 ( 65.9) |
|  | Yes | 810 ( 34.2) | 1217 ( 31.5) | 1792 ( 29.4) | 769 ( 37.8) | 1131 ( 35.4) | 1548 ( 34.1) |
| **Personality** | |  |  |  |  |  |  |
|  | No | 2354 ( 99.5) | 3841 ( 99.4) | 6069 ( 99.5) | 2022 ( 99.4) | 3169 ( 99.3) | 4512 ( 99.3) |
|  | Yes | 13 ( 0.5) | 23 ( 0.6) | 31 ( 0.5) | 12 ( 0.6) | 23 ( 0.7) | 30 ( 0.7) |
| **Schizophrenia** | |  |  |  |  |  |  |
|  | No | 2025 ( 85.6) | 3376 ( 87.4) | 5472 ( 89.7) | 1746 ( 85.8) | 2798 ( 87.7) | 4089 ( 90.0) |
|  | Yes | 342 ( 14.4) | 488 ( 12.6) | 628 ( 10.3) | 288 ( 14.2) | 394 ( 12.3) | 453 ( 10.0) |
| **Alcohol** |  |  |  |  |  |  |  |
|  | No | 1959 ( 82.8) | 3147 ( 81.4) | 4940 ( 81.0) | 1671 ( 82.2) | 2573 ( 80.6) | 3616 ( 79.6) |
|  | Yes | 408 ( 17.2) | 717 ( 18.6) | 1160 ( 19.0) | 363 ( 17.8) | 619 ( 19.4) | 926 ( 20.4) |
| **Substance** | |  |  |  |  |  |  |
|  | No | 1999 ( 84.5) | 3243 ( 83.9) | 5109 ( 83.8) | 1723 ( 84.7) | 2674 ( 83.8) | 3764 ( 82.9) |
|  | Yes | 368 ( 15.5) | 621 ( 16.1) | 991 ( 16.2) | 311 ( 15.3) | 518 ( 16.2) | 778 ( 17.1) |
| **Suicide** |  |  |  |  |  |  |  |
|  | No | 2297 ( 97.0) | 3770 ( 97.6) | 5967 ( 97.8) | 1967 ( 96.7) | 3106 ( 97.3) | 4428 ( 97.5) |
|  | Yes | 70 ( 3.0) | 94 ( 2.4) | 133 ( 2.2) | 67 ( 3.3) | 86 ( 2.7) | 114 ( 2.5) |
| **Screening** | |  |  |  |  |  |  |
|  | No | 2329 ( 98.4) | 3795 ( 98.2) | 5984 ( 98.1) | 2002 ( 98.4) | 3138 ( 98.3) | 4463 ( 98.3) |
|  | Yes | 38 ( 1.6) | 69 ( 1.8) | 116 ( 1.9) | 32 ( 1.6) | 54 ( 1.7) | 79 ( 1.7) |
| **Miscellaneous** | |  |  |  |  |  |  |
|  | No | 2338 ( 98.8) | 3807 ( 97.5) | 5992 ( 98.2) | 2015 ( 99.1) | 3157 ( 98.9) | 4491 ( 98.9) |
|  | Yes | 29 ( 1.2) | 57 ( 1.5) | 108 ( 1.8) | 19 ( 0.9) | 35 ( 1.1) | 51 ( 1.1) |
