# Supplementary material for: Predictors of Return Visits Among Insured Emergency Department Mental Health and Substance Abuse Patients, 2005–2013
Source: West J Emerg Med. 2017 Jul 17;18(5):884–93. doi: 10.5811/westjem.2017.6.33850 (PMC5576625; doi:10.5811/westjem.2017.6.33850)
Supplement: Supplementary file 4 [file wjem-18-884-s004.docx]

| **Supplemental Table 2a.** Logistic regression analysis showing rates of 3 day, 7 day, and 30 day return ED visit by patient characteristics. | | | | | | |  |  |
| --- | --- | --- | --- | --- | --- | --- | --- | --- |
|  |  |  |  |  |  | |  | |
|  | **All returns** | | | **MHSA returns** | | | | |
|  | **3day** | **7day** | **30day** | **3day** | | **7day** | | **30day** |
| **Characteristic** | **OR** | **OR** | **OR** | **OR** | | **OR** | | **OR** |
| **Sex** |  |  |  |  | |  | |  |
| Female | ref | ref | ref | Ref | | Ref | | Ref |
| Male | 1.08 [1.03,1.13] | 1.09 [1.05,1.12] | 1.07 [1.05,1.09] | 1.12 [1.06,1.19] | | 1.15 [1.10,1.21] | | 1.18 [1.14,1.22] |
| **Age (category)** |  |  |  |  | |  | |  |
| <18 | ref | ref | ref | Ref | | Ref | | Ref |
| 18-35 | **1.48 [1.36,1.62]** | **1.38 [1.30,1.47]** | **1.19 [1.15,1.24]** | **1.38 [1.23,1.54]** | | **1.22 [1.13,1.32]** | | **0.99 [0.94,1.05]** |
| 36-64 | **1.85 [1.69,2.02]** | **1.68 [1.58,1.79]** | **1.37 [1.32,1.42]** | **1.69 [1.51,1.90]** | | **1.49 [1.38,1.62]** | | **1.15 [1.09,1.21]** |
| >65 | **2.37 [2.12,2.65]** | **2.17 [2.00,2.34]** | **1.80 [1.71,1.90]** | **2.10 [1.80,2.44]** | | **1.77 [1.59,1.97]** | | **1.27 [1.18,1.37]** |
| **Hwang** |  |  |  |  | |  | |  |
| 0 | ref | ref | ref | Ref | | Ref | | Ref |
| 1 | 0.97 [0.90,1.05] | 0.99 [0.94,1.05] | 0.90 [0.87,0.94] | 1.03 [0.93,1.15] | | 1.05 [0.97,1.14] | | 0.99 [0.94,1.05] |
| 2 | 0.95 [0.88,1.02] | 1.02 [0.97,1.08] | 0.99 [0.96,1.03] | 0.99 [0.89,1.10] | | **1.13 [1.04,1.22]** | | **1.14 [1.08,1.20]** |
| 3 | **1.09 [1.01,1.18]** | **1.11 [1.05,1.17]** | **1.10 [1.06,1.14]** | **1.15 [1.04,1.28]** | | **1.20 [1.11,1.30]** | | **1.23 [1.17,1.30]** |
| 4 | 1.05 [0.97,1.15] | **1.12 [1.06,1.19]** | **1.11 [1.07,1.16]** | **1.16 [1.04,1.31]** | | **1.26 [1.16,1.38]** | | **1.29 [1.21,1.37]** |
| 5+ | **1.11 [1.04,1.19]** | **1.18 [1.13,1.24]** | **1.21 [1.17,1.25]** | 1.08 [0.98,1.19] | | **1.17 [1.09,1.26]** | | **1.18 [1.12,1.24]** |
| **Prior EDs** |  |  |  |  | |  | |  |
| 0 | Ref | ref | ref | Ref | | Ref | | Ref |
| 1 | **1.41 [1.33,1.49]** | **1.47 [1.41,1.53]** | **1.58 [1.54,1.62]** | **1.31 [1.21,1.41]** | | **1.34 [1.27,1.42]** | | **1.35 [1.30,1.41]** |
| 2 | **1.77 [1.65,1.90]** | **1.91 [1.82,2.01]** | **2.20 [2.12,2.27]** | **1.47 [1.33,1.63]** | | **1.53 [1.42,1.65]** | | **1.63 [1.55,1.71]** |
| 3 | **2.28 [2.08,2.49]** | **2.53 [2.38,2.69]** | **3.04 [2.92,3.17]** | **1.86 [1.64,2.11]** | | **1.92 [1.75,2.11]** | | **1.97 [1.85,2.10]** |
| 4+ | **3.73 [3.49,3.98]** | **4.31 [4.11,4.52]** | **5.94 [5.75,6.14]** | **2.29 [2.07,2.54]** | | **2.41 [2.24,2.60]** | | **2.53 [2.40,2.67]** |
| **Initial visit**  **CCS category** | |  |  |  | |  | |  |
| **Adjustment** |  |  |  |  | |  | |  |
| No | Ref | ref | ref | Ref | | Ref | | Ref |
| Yes | 1.23 [1.04,1.44] | 1.17 [1.05,1.32] | 1.13 [1.04,1.22] | 1.54 [1.25,1.89] | | 1.55 [1.33,1.79] | | 1.36 [1.22,1.51] |
| **Anxiety** |  |  |  |  | |  | |  |
| No | Ref | ref | ref | Ref | | Ref | | Ref |
| Yes | 1.02 [0.93,1.12] | 1.00 [0.93,1.06] | **0.91 [0.87,0.95]** | 1.01 [0.89,1.14] | | 1.01 [0.93,1.11] | | **0.91 [0.85,0.97]** |
| **ADHD** |  |  |  |  | |  | |  |
| No | Ref | ref | ref | Ref | | Ref | | Ref |
| Yes | 1.25 [1.06,1.48] | 1.18 [1.05,1.32] | 1.05 [0.97,1.14] | 1.62 [1.33,1.99] | | 1.63 [1.41,1.88] | | 1.41 [1.28,1.55] |
| **D/O Childhood** |  |  |  |  | |  | |  |
| No | Ref | ref | ref | Ref | | Ref | | Ref |
| Yes | 1.32 [0.90,1.93] | 1.03 [0.78,1.38] | 1.08 [0.90,1.28] | 1.56 [0.96,2.51] | | 1.25 [0.87,1.81] | | 1.43 [1.15,1.79] |
| **Impulse** |  |  |  |  | |  | |  |
| No | Ref | ref | ref | Ref | | Ref | | Ref |
| Yes | 0.96 [0.54,1.70] | 0.91 [0.61,1.37] | 1.09 [0.86,1.38] | 1.25 [0.62,2.53] | | 1.38 [0.86,2.22] | | 1.72 [1.29,2.28] |
| **Mood** |  |  |  |  | |  | |  |
| No | Ref | ref | ref | Ref | | Ref | | Ref |
| Yes | **1.25 [1.14,1.36]** | **1.25 [1.17,1.33]** | **1.18 [1.13,1.24]** | **1.89 [1.68,2.12]** | | **1.93 [1.77,2.10]** | | **1.83 [1.73,1.95]** |
| **Personality** |  |  |  |  | |  | |  |
| No | Ref | ref | ref | Ref | | Ref | | Ref |
| Yes | **1.75 [1.24,2.45]** | **1.58 [1.23,2.03]** | **1.54 [1.29,1.83]** | **2.08 [1.35,3.20]** | | **1.90 [1.37,2.61]** | | **1.92 [1.55,2.38]** |
| **Schizophrenia** |  |  |  |  | |  | |  |
| No | Ref | ref | ref | Ref | | Ref | | Ref |
| Yes | **1.41 [1.25,1.58]** | **1.32 [1.21,1.43]** | **1.16 [1.09,1.23]** | **1.72 [1.48,2.00]** | | **1.62 [1.45,1.82]** | | **1.48 [1.37,1.61]** |
| **Alcohol** |  |  |  |  | |  | |  |
| No | Ref | ref | ref | Ref | | Ref | | Ref |
| Yes | **0.66 [0.59,0.73]** | **0.60 [0.55,0.65]** | **0.56 [0.53,0.59]** | **0.75 [0.65,0.86]** | | **0.68 [0.61,0.75]** | | **0.68 [0.64,0.74]** |
| **Substance** |  |  |  |  | |  | |  |
| No | Ref | ref | ref | Ref | | Ref | | Ref |
| Yes | **1.27 [1.15,1.41]** | **1.16 [1.08,1.26]** | **1.03 [0.98,1.08]** | **1.44 [1.25,1.65]** | | **1.23 [1.11,1.37]** | | **1.05 [0.97,1.13]** |
| **Suicide** |  |  |  |  | |  | |  |
| No | Ref | ref | ref | Ref | | Ref | | Ref |
| Yes | **0.68 [0.55,0.84]** | **0.69 [0.59,0.80]** | **0.84 [0.77,0.92]** | **0.72 [0.54,0.96]** | | **0.78 [0.64,0.95]** | | 0.94 [0.84,1.07] |
| **Screening** |  |  |  |  | |  | |  |
| No | Ref | ref | ref | Ref | | Ref | | Ref |
| Yes | 1.07 [0.90,1.27] | 1.08 [0.96,1.22] | 1.03 [0.95,1.12] | 0.89 [0.68,1.16] | | 0.91 [0.75,1.10] | | 0.99 [0.87,1.12] |
| **Miscellaneous** |  |  |  |  | |  | |  |
| No | Ref | ref | ref | Ref | | Ref | | Ref |
| Yes | 1.30 [1.13,1.49] | 1.28 [1.16,1.42] | 1.13 [1.06,1.22] | 0.81 [0.64,1.03] | | 0.87 [0.74,1.03] | | 0.77 [0.69,0.87] |
