# Supplementary material for: Predictors of Return Visits Among Insured Emergency Department Mental Health and Substance Abuse Patients, 2005–2013
Source: West J Emerg Med. 2017 Jul 17;18(5):884–93. doi: 10.5811/westjem.2017.6.33850 (PMC5576625; doi:10.5811/westjem.2017.6.33850)
Supplement: Supplementary file 5 [file wjem-18-884-s005.docx]

| **Supplemental Table 2b.** Logistic regression analysis showing rates of 3 day, 7 day, and 30 day return hospitalization by patient characteristics. | | | | | | | |
| --- | --- | --- | --- | --- | --- | --- | --- |
|  | **All returns** | | | | **MHSA returns** | | |
|  | **3day** | **7day** | **30day** | | **3day** | **7day** | **30day** |
| **Characteristic** | **OR** | **OR** | **OR** | | **OR** | **OR** | **OR** |
| **Age (category)** |  |  |  | |  |  |  |
| <18 | Ref | Ref | Ref | | Ref | ref | Ref |
| 18-35 | 1.19 [1.03,1.38] | 0.97 [0.87,1.08] | 0.86 [0.79,0.94] | | 1.18 [1.02,1.37] | 0.95 [0.85,1.07] | 0.80 [0.73,0.88] |
| 36-64 | 1.51 [1.31,1.74] | 1.26 [1.13,1.41] | 1.14 [1.05,1.25] | | 1.38 [1.19,1.60] | 1.13 [1.01,1.27] | 0.95 [0.87,1.05] |
| >65 | 1.06 [0.86,1.31] | 0.84 [0.72,0.99] | 0.81 [0.71,0.92] | | 0.92 [0.73,1.16] | 0.66 [0.54,0.79] | 0.49 [0.41,0.57] |
| **Sex** |  |  |  | |  |  |  |
| Female | ref | Ref | Ref | | Ref | ref | Ref |
| Male | 1.11 [1.02,1.21] | 1.11 [1.04,1.18] | 1.10 [1.04,1.16] | | 1.17 [1.07,1.28] | 1.16 [1.08,1.25] | 1.15 [1.08,1.22] |
| **Hwang** |  |  |  | |  |  |  |
| 0 | ref | Ref | Ref | | Ref | ref | Ref |
| 1 | 0.89 [0.76,1.03] | 0.81 [0.71,0.92] | 0.83 [0.76,0.92] | | 0.88 [0.75,1.04] | 0.80 [0.70,0.91] | 0.83 [0.74,0.93] |
| 2 | **1.16 [1.01,1.34]** | **1.23 [1.10,1.38]** | **1.24 [1.13,1.36]** | | 1.12 [0.96,1.31] | **1.19 [1.05,1.34]** | **1.24 [1.12,1.37]** |
| 3 | **1.38 [1.19,1.59]** | **1.46 [1.31,1.64]** | **1.54 [1.40,1.68]** | | **1.37 [1.18,1.60]** | **1.45 [1.29,1.64]** | **1.55 [1.40,1.72]** |
| 4 | **1.57 [1.35,1.83]** | **1.70 [1.51,1.92]** | **1.68 [1.53,1.86]** | | **1.49 [1.26,1.75]** | **1.61 [1.41,1.83]** | **1.58 [1.41,1.77]** |
| 5+ | **1.43 [1.26,1.64]** | **1.67 [1.51,1.86]** | **1.85 [1.70,2.01]** | | **1.31 [1.13,1.51]** | **1.48 [1.32,1.66]** | **1.54 [1.39,1.70]** |
| **Prior EDs** |  |  |  | |  |  |  |
| 0 | ref | Ref | Ref | | Ref | ref | ref |
| 1 | **1.26 [1.14,1.40]** | **1.22 [1.12,1.32]** | **1.27 [1.19,1.36]** | | **1.20 [1.08,1.34]** | **1.16 [1.06,1.27]** | **1.23 [1.14,1.32]** |
| 2 | **1.55 [1.36,1.76]** | **1.57 [1.42,1.74]** | **1.54 [1.42,1.67]** | | **1.51 [1.31,1.73]** | **1.49 [1.33,1.67]** | **1.42 [1.29,1.57]** |
| 3 | **1.58 [1.33,1.88]** | **1.67 [1.46,1.92]** | **1.66 [1.49,1.85]** | | **1.49 [1.23,1.81]** | **1.60 [1.38,1.86]** | **1.53 [1.35,1.75]** |
| 4+ | **1.59 [1.37,1.84]** | **1.79 [1.60,2.01]** | **1.66 [1.51,1.82]** | | **1.46 [1.24,1.72]** | **1.63 [1.44,1.86]** | **1.42 [1.26,1.59]** |
| **Initial visit**  **CCS category** | |  |  | |  |  |  |
| **Adjustment** |  |  |  | |  |  |  |
| No | ref | ref | Ref | | Ref | ref | ref |
| Yes | 0.73 [0.50,1.06] | 0.79 [0.59,1.05] | 0.79 [0.62,0.99] | | 0.82 [0.56,1.21] | 0.89 [0.66,1.20] | 0.85 [0.66,1.10] |
| **Anxiety** |  |  |  | |  |  |  |
| No | ref | ref | Ref | | Ref | ref | Ref |
| Yes | **0.52 [0.44,0.62]** | **0.62 [0.55,0.71]** | **0.68 [0.61,0.75]** | | **0.45 [0.38,0.54]** | **0.52 [0.45,0.60]** | **0.53 [0.47,0.60]** |
| **ADHD** |  |  |  | |  |  |  |
| No | ref | ref | Ref | | Ref | ref | Ref |
| Yes | 1.80 [1.40,2.31] | 1.87 [1.54,2.26] | 1.60 [1.37,1.88] | | 1.99 [1.54,2.58] | 1.96 [1.61,2.40] | 1.73 [1.46,2.05] |
| **D/O Childhood** |  |  |  | |  |  |  |
| No | Ref | ref | Ref | | Ref | ref | ref |
| Yes | 0.96 [0.45,2.05] | 1.60 [1.01,2.52] | 1.76 [1.25,2.47] | | 0.92 [0.41,2.09] | 1.72 [1.08,2.74] | 1.77 [1.23,2.56] |
| **Impulse** |  |  |  | |  |  |  |
| No | Ref | ref | Ref | | Ref | ref | ref |
| Yes | 1.30 [0.53,3.17] | 0.94 [0.42,2.11] | 1.53 [0.92,2.53] | | 1.48 [0.61,3.60] | 1.08 [0.48,2.43] | 1.76 [1.04,2.95] |
| **Mood** |  |  |  | |  |  |  |
| No | Ref | ref | Ref | | Ref | ref | ref |
| Yes | **2.20 [1.89,2.55]** | **2.07 [1.84,2.33]** | **1.82 [1.66,2.01]** | | **2.61 [2.23,3.05]** | **2.47 [2.18,2.80]** | **2.24 [2.01,2.49]** |
| **Personality** |  |  |  | |  |  |  |
| No | Ref | ref | Ref | | Ref | ref | ref |
| Yes | **1.96 [1.11,3.45]** | **1.95 [1.27,3.01]** | **1.54 [1.06,2.24]** | | **2.04 [1.13,3.67]** | **2.27 [1.47,3.50]** | **1.89 [1.29,2.76]** |
| **Schizophrenia** |  |  |  | |  |  |  |
| No | Ref | ref | Ref | | Ref | ref | ref |
| Yes | **3.07 [2.58,3.67]** | **2.84 [2.47,3.27]** | **2.21 [1.96,2.49]** | | **3.31 [2.75,3.98]** | **3.09 [2.66,3.60]** | **2.54 [2.21,2.90]** |
| **Alcohol** |  |  |  | |  |  |  |
| No | Ref | ref | Ref | | Ref | ref | ref |
| Yes | **0.91 [0.77,1.09]** | 1.08 [0.94,1.23] | 1.06 [0.95,1.18] | | 0.98 [0.82,1.18] | 1.15 [0.99,1.33] | 1.15 [1.02,1.30] |
| **Substance** |  |  |  | |  |  |  |
| No | Ref | ref | Ref | | Ref | ref | ref |
| Yes | **1.76 [1.48,2.10]** | **1.93 [1.69,2.21]** | **1.90 [1.70,2.12]** | | **1.83 [1.52,2.20]** | **2.03 [1.76,2.35]** | **2.11 [1.86,2.38]** |
| **Suicide** |  |  |  | |  |  |  |
| No | Ref | Ref | Ref | | Ref | ref | ref |
| Yes | **1.88 [1.47,2.41]** | **1.59 [1.28,1.97]** | **1.40 [1.17,1.67]** | | **2.05 [1.59,2.64]** | **1.70 [1.36,2.12]** | **1.52 [1.25,1.85]** |
| **Screening** |  |  |  | |  |  |  |
| No | ref | Ref | Ref | | Ref | ref | ref |
| Yes | 0.97 [0.69,1.37] | 1.15 [0.89,1.48] | 1.21 [0.99,1.48] | | 0.98 [0.68,1.42] | 1.11 [0.84,1.48] | 1.12 [0.89,1.42] |
| **Miscellaneous** |  |  |  |  | |  |  |
| No | ref | Ref | Ref | Ref | | ref | ref |
| Yes | 0.54 [0.37,0.80] | 0.67 [0.51,0.89] | 0.78 [0.63,0.96] | 0.45 [0.28,0.71] | | 0.54 [0.38,0.76] | 0.53 [0.40,0.71] |
