# Supplementary material for: Predictors of Return Visits Among Insured Emergency Department Mental Health and Substance Abuse Patients, 2005–2013
Source: West J Emerg Med. 2017 Jul 17;18(5):884–93. doi: 10.5811/westjem.2017.6.33850 (PMC5576625; doi:10.5811/westjem.2017.6.33850)
Supplement: Supplementary file 6 [file wjem-18-884-s006.docx]

**Supplemental Figure.** Distribution of the return visits.
